# γKlotho is a novel marker and cell survival factor in a subset of triple negative breast cancers

**DOI:** 10.18632/oncotarget.6006

**Published:** 2015-11-09

**Authors:** Nuša Trošt, Samuel Peña-Llopis, Sajjan Koirala, Jurij Stojan, Patrick Ryan Potts, Klementina Fon Tacer, Elisabeth D. Martinez

**Affiliations:** ^1^ Institute of Biochemistry, Faculty of Medicine, University of Ljubljana, Ljubljana, Slovenia; ^2^ Department of Pharmacology, University of Texas Southwestern Medical Center, Dallas, Texas, USA; ^3^ Department of Physiology, University of Texas Southwestern Medical Center, Dallas, Texas, USA; ^4^ Hamon Center for Therapeutic Oncology Research, University of Texas Southwestern Medical Center, Dallas, Texas, USA

**Keywords:** TNBC, cancer therapy, Klotho, FGF, oxidative stress

## Abstract

Over the last decade, breast cancer mortality has declined. However, triple negative breast cancer (TNBC) remains a challenging problem mostly due to early recurrence and lack of molecularly driven treatments. There is a critical need to identify subgroups of TNBC with common molecular features that can be therapeutically targeted. Here we show that in contrast to Klotho and βKlotho, the third member of the Klotho protein family, γKlotho, is overexpressed in more than 60% of TNBCs and correlates with poorer disease progression. Furthermore, we find that γKlotho is expressed in a subset of TNBC cell lines promoting cell growth. Importantly, we demonstrate that in these cells γKlotho is necessary for cell survival and that its depletion leads to constitutive ERK activation, cell cycle arrest and apoptosis. Interestingly, we observe increased oxidative stress in γKlotho-depleted cells suggesting that γKlotho enables cancer cells to cope with an oxidative environment and that cells become dependent on its expression to maintain this survival advantage. These findings indicate that γKlotho might be a potential marker for patients that would benefit from treatments that alter oxidative stress and constitutes a novel drug target for a subset of TN breast cancers.

## INTRODUCTION

Due to improved detection and therapy, breast cancer mortality has declined since 1990. However, it still remains the leading cause of cancer related deaths among women [1, 2]. In particular, triple negative breast cancers (TNBCs) defined as lacking estrogen and progesterone receptor expression and not harboring amplification of human epidermal growth factor receptor 2 (HER2), account for a disproportionate number of deaths from breast cancer and represent around 15–20% of newly diagnosed cases [3, 4]. TNBC tumors are generally larger in size, of higher histological grade and more aggressive with poor prognosis [5–7]. Fewer than 30% of women with metastatic TNBC survive 5 years, and almost all die of their disease despite adjuvant chemotherapy, the only therapy currently available for these patients [8]. To develop new strategies against this aggressive type of breast cancer, we need to gain a better understanding of the molecular events driving TNBC tumorigenesis.

Malignant transformation is frequently associated with enhanced cellular oxidative stress. Reactive oxygen species (ROS) mediate growth factor signaling [9–11] and contribute to malignant phenotypes [12, 13] by stimulating cell growth and proliferation, promoting genetic instability and helping evade senescence [14–16]. At the same time, high levels of and prolonged exposure to ROS can cause cellular damage. Therefore, the antioxidant system is critically important and often amplified in cancer [17, 18]. In addition, cancer cells may be more vulnerable to oxidant stress as they function at a heightened basal level of ROS.

Klotho, the co-receptor for endocrine fibroblast growth factors, was originally found to decrease oxidative stress and prevent aging [19, 20]. The Klotho family comprises 3 proteins. Klotho and βKlotho function as obligate co-receptors for endocrine fibroblast growth factors whereas the physiological function of the third member, γKlotho (referred to also as Lctl), remains unknown [21–23]. We have recently shown that γKlotho also binds to FGF receptors (FGFR) [24]. FGF and FGFRs are proto-oncogenes often activated in several human cancers [25, 26]. For example, FGFR-1 and/or FGFs are frequently found amplified in breast cancer [27, 28]. FGFR-2 polymorphisms have been identified as major breast cancer risk factors in genome-wide association studies [29]. In contrast to FGFs and FGFRs, Klothos are epigenetically silenced in several cancers and have tumor-suppressor activity [30–34]. Here we found that in contrast to the *bona fide* Klotho proteins, γKlotho is upregulated in breast cancer compared to benign patient-matched tissue. In particular, γKlotho is highly expressed in a subset of TNBC patients where Klotho and βKlotho are significantly downregulated. We show that γKlotho is necessary for TNBC cell survival in an FGF independent manner and that its depletion leads to increased oxidative stress, DNA damage, and cell death. Our results suggest that γKlotho may be a prospective drug target for the treatment of a subset of TNBC patients and a bio-marker for patients that might benefit from anticancer agents inducing oxidative stress.

## RESULTS

### γKlotho is upregulated in a subset of triple negative breast cancers

**Figure 1:**
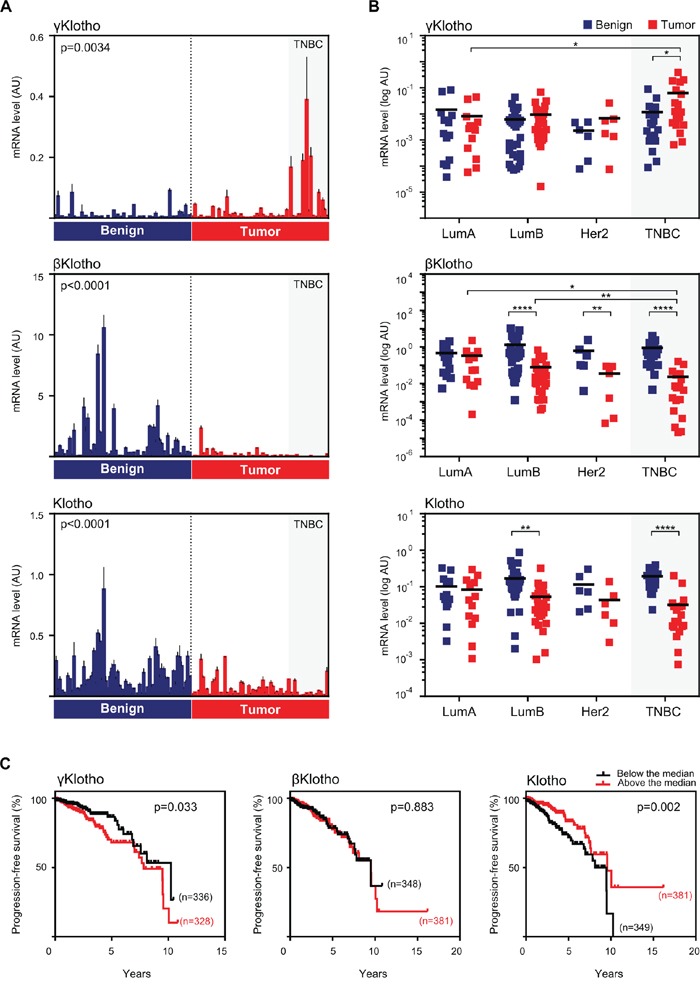
γKlotho is up-regulated in human triple negative breast cancer **A.** The expression of Klotho genes in normal/benign breast tissue (blue bars) and breast cancer (red bars). 68 samples of breast cancer specimens with corresponding patient-matched normal breast tissue were analyzed for mRNA expression of γKlotho, βKlotho, and Klotho by qRT-PCR. Expression levels were normalized against cyclophilin B. Each bar represents the mean ± SD of three replicates. Statistical analysis was performed on log-transformed data. Tumor and control groups were compared by paired *t*-test. *p* < 0.05 was considered statistically significant. **B.** In triple negative breast cancers γKlotho shows the opposite gene expression pattern than βKlotho and Klotho. Paired (benign and tumor) samples were divided into four groups according to the molecular subtype: luminal A (LumA; *n* = 13), luminal B (LumB; *n* = 30), HER2 (Her2; *n* = 6) and triple-negative breast cancer (TNBC; *n* = 19). The difference in gene expression between the subgroups was tested for significance using a two-way ANOVA followed by Bonferroni post-hoc tests on log-transformed data. Individual mRNA levels are presented on scatter dot plots using logarithmic scale for the y-axis. Black line denotes the mean. *p ≤ 0.05, **p.< 0.01, ***p ≤ 0.001, ****p ≤ 0.0001. **C.** Kaplan-Meier progression-free survival curves according to the expression level with respect to the median of each Klotho gene in patients with invasive breast carcinoma with available triple negative status. Clinical and gene expression data were obtained from the TCGA portal. Log-rank (Mantel-Cox) tests were used to compare groups. Censored subjects are indicated on the curves by tick marks.

To determine the function of the third member of the Klotho family, γKlotho, in cancer we first examined mRNA expression of all three Klotho genes in sixty eight paired samples of tumor and benign tissue from breast cancer patients, and analyzed gene expression patterns in relation to clinical parameters and molecular subtypes (Figure 1 and Supplementary Table S1). Consistent with previous findings [30, 31], we found that Klotho is downregulated in breast cancer samples compared to benign controls (Figure 1A). In addition to Klotho, we also found significant downregulation of βKlotho expression in breast cancer specimens. Interestingly, γKlotho showed the opposite pattern of expression and was significantly upregulated in cancer relative to normal breast tissue (Figure 1A). Strikingly, the majority of samples with high γKlotho expression classified as triple negative breast tumors (TNBC) (Figure 1A). Thus, we further analyzed the gene expression data grouped into four major breast cancer molecular subtypes, luminal A, luminal B, HER2 type and triple negative. It became evident that the three Klotho genes are differentially expressed specifically in the triple negative tumors, where γKlotho is significantly upregulated (in 13/19 TN samples) as Klotho and βKlotho are downregulated (Figure 1B). Furthermore, we found that γKlotho expression in tumors correlated positively with Ki67 proliferative index (Table 1), suggesting a potential role in more aggressive/higher stage breast cancers. This indicates that the three Klothos have distinct functions in tumorigenesis consistent with differences in their protein structure (Supplementary Figure S1A).

**Table 1:** Correlation between expression levels of Klotho genes (determined by qRT-PCR) and Ki67/p53 prognostic expression levels (determined by IHC) in 67 tumor samples

	γKlotho		βKlotho		Klotho	
	Spearman *r*	*q*-value	Spearman *r*	*q*-value	Spearman *r*	*q*-value
**Ki67**	0.31	**0.024**	−0.44	**0.0006**	−0.13	0.34
**p53**	0.062	0.62	−0.48	**< 0.0001**	−0.22	0.11

Significant FDR-corrected *p*-values (*q*-values) are given in bold (Spearman rank correlation test, Benjamini and Hochberg false discovery rate (FDR) correction).

To validate these results, we analyzed The Cancer Genome Atlas (TCGA) [35, 36] and the Curtis datasets [37] of breast malignancies and confirmed that γKlotho is significantly overexpressed in TN tumors also in these two patient collections (Supplementary Figure S1B and S1C). In line with these results, we found that patients with higher expression of γKlotho in the breast cancer population have a significant decrease in progression-free survival (TCGA dataset, Figure 1C) and that γKlotho expression correlates with higher grade and stage (Curtis dataset, Supplementary Table S2). In our dataset, we also found a significant correlation between higher γKlotho expression and tumor grade (Supplementary Table S2). In contrast to high γKlotho expression, patients with tumors expressing high levels of Klotho showed increased progression-free survival and overall survival (Supplementary Figure S1C and S1D).

To determine whether this expression pattern of Klothos in normal vs. cancer tissue is specific for breast cancer, we analyzed expression data in tumors and corresponding normal tissue of different cancer types in TCGA database (Supplementary Figure S1E). Interestingly, we found increased expression of γKlotho accompanied with decreased expression of Klotho and βKlotho in several other cancers, including glioblastoma multiforme, lung squamous and lung adenocarcinomas but not in liver hepatocellular carcinoma. Together, our results demonstrate that γKlotho is overexpressed in a subset of TN breast tumors where Klotho and βKlotho are downregulated. In addition, γKlotho expression levels correlate with higher Ki-67, worse progression-free survival and higher grade/stage of disease. Our data suggest that γKlotho may represent a novel marker and potential oncogene in a subgroup of triple negative breast tumors.

### γKlotho is expressed in a subgroup of TNBC cell lines and its overexpression increases cell viability and clonogenic growth

**Figure 2:**
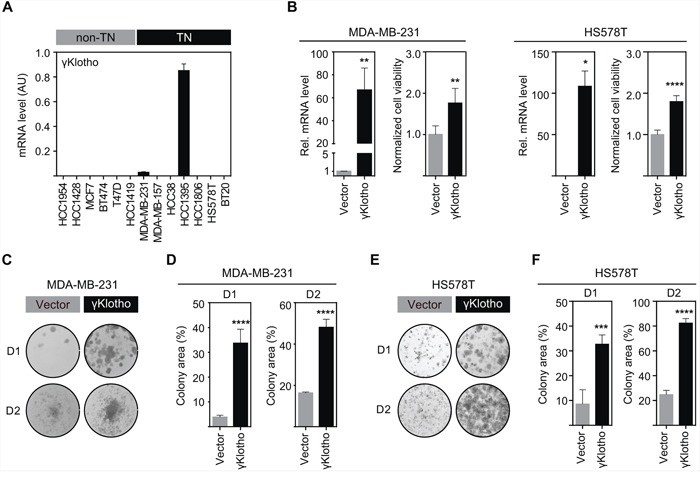
γKlotho is expressed in a subset of triple negative cell lines and its overexpression promotes cell viability and colony formation of triple negative MDA-MB-231 and HS578T cells **A.** γKlotho mRNA expression was determined by qRT-PCR and normalized against Rplp0. Each bar represents the mean ± SD of two biological replicates. **B.** γKlotho overexpression promotes cell viability. MDA-MB-231 and HS578T cells stably overexpressing γKlotho were assayed for viability by MTS assay on day 4 after seeding. Viability 12 hours post seeding was used for normalization. Data are presented as mean ± SD of 6 wells from one representative experiment. Viability experiments were repeated at least three times for each cell line. *p ≤ 0.05 **p ≤ 0.01, ****p ≤ 0.0001; Unpaired t test. γKlotho mRNA levels were determined by qRT-PCR and are presented relative to control (vector). **C, E.** γKlotho overexpression promotes liquid colony formation. MDA-MB-231/HS578T cells were seeded in triplicates in 12-well plates at two different densities (D1=1000/500 cells/well, D2=5000/2500 cells/well). Ten (D2) and fourteen (D1) days after plating, colonies were stained with crystal violet and photographed. Representative images of 3 independent experiments are shown. **D, F.** Cell growth was quantified by determining the percentage of area covered by crystal violet stained cell colonies. Data are presented as mean of three replicates ± SD for each density. ***p ≤ 0.001, ****p ≤ 0.0001; two way ANOVA.

To investigate the role of γKlotho in cell viability and on the oncogenic potential of breast cancer cells, we first examined the expression of γKlotho at the mRNA level in a panel of various breast cancer cell lines (Figure 2A). Consistent with our patient data (Figure 1A and 1B), we found that γKlotho is expressed in TNBC cell lines HCC1395 and MDA-MB-231, but not in any non-TN line (Figure 2A). Klotho and βKlotho were not expressed in the γKlotho expressing cells (Supplementary Figure S2A and S2B). To determine whether γKlotho can be an oncogenic driver in TNBC, we overexpressed γKlotho in MDA-MB-231 and HS578T cells (Figure 2B) and assayed the effect of γKlotho overexpression on cell viability and clonogenic potential. γKlotho overexpression significantly increased viability of cells as measured by MTS assay in both cell lines (Figure 2B). Additionally, γKlotho overexpression stimulated the ability of cells to form colonies on a solid surface (Figure 2C–2F) independent of starting density and promoted growth in soft agar (Supplementary Figure S2C and S2D). Thus, γKlotho is expressed in a subset of triple negative breast cancer cell lines and when overexpressed promotes cell viability and drives colony formation indicative of features of an oncogene.

### γKlotho is required for survival and oncogenic growth of triple negative breast cancer cells

**Figure 3:**
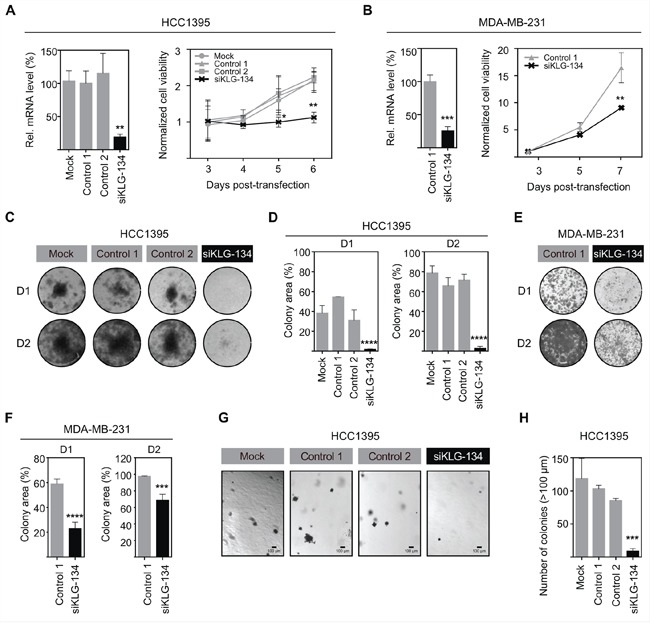
γKlotho is necessary for TNBC cell survival and clonogenic growth **A.** siRNA knockdown of γKlotho decreases the viability of HCC1395 cells. γKlotho expression in TNBC HCC1395 cells was knocked down by a pool of 3 siRNAs (siKLG-134) and the efficiency of γKlotho knockdown confirmed by qRT-PCR. mRNA levels are presented relative to control. **p ≤ 0.01; one-way ANOVA. 48 hours after transfection, cells were seeded and cell viability was monitored for 4 days by MTS assay. 12 hours post seeding-viability was used for normalization. Means of three independent experiments ± SD are presented. *p ≤ 0.05 **p ≤ 0.01; two-way ANOVA. **B.** siRNA knockdown of γKlotho decreases the viability of MDA-MB-231 cells. Mean of two independent experiments ± SD are presented. **p ≤ 0.01; two-way ANOVA. **C, E.** Knockdown of γKlotho inhibits liquid colony formation of HCC1395 and MDA-MB-231 cells. Cells were seeded in triplicates in 12-well plates at two different densities (D1=1000cells/well, D2=5000 cells/well). Ten (D2) and fourteen (D1) days after plating, colonies were stained with crystal violet and photographed. Representative images are shown. **D, F.** Liquid colony formation was quantified by determining the percentage of area covered by crystal violet stained cell colonies using ImageJ ColonyArea plugin. Data are presented as mean ± SD of three replicates. ***p ≤ 0.001, ****p ≤ 0.0001; two-way ANOVA. **G.** Knockdown of γKlotho inhibits the anchorage-independent growth on soft agar of HCC1395 cells. Cells were transfected with a siRNA pool against γKlotho (siKLG-134) and corresponding controls and after 48 hours plated for anchorage-independent growth in soft agar. Representative images for each condition are presented. **H.** Colonies greater than 100 μm across their widest point were counted. Bars demonstrate average ± SD across triplicates. ***p ≤ 0.001, one-way ANOVA.

To examine if γKlotho is necessary for the viability of HCC1395 and MDA-MB-231 TNBC cells, we specifically depleted γKlotho expression by siRNA. Four siRNAs against different regions of the γKlotho gene were tested, and a pool of the three most efficient ones (1, 3, and 4) was then used in subsequent experiments (Figures 3A and 3B). Knockdown of γKlotho significantly decreased viability of both cell lines as evaluated over time by MTS assay (Figure 3A and 3B, right panels) while not altering the expression of other Klotho genes, which remained undetectable. Furthermore, γKlotho knockdown resulted in a significant reduction of the ability of tumor cells to form colonies on a solid surface (Figure 3C–3F) and of anchorage independent growth in soft agar (Figure 3G and 3H). Together, our results show that γKlotho is necessary for the survival of γKlotho positive triple negative breast cancer cells implying that these cells become at least partly dependent on its expression.

### The growth inhibitory effect of γKlotho knockdown is specific and can be partially rescued by an siRNA-resistant mouse gene

**Figure 4:**
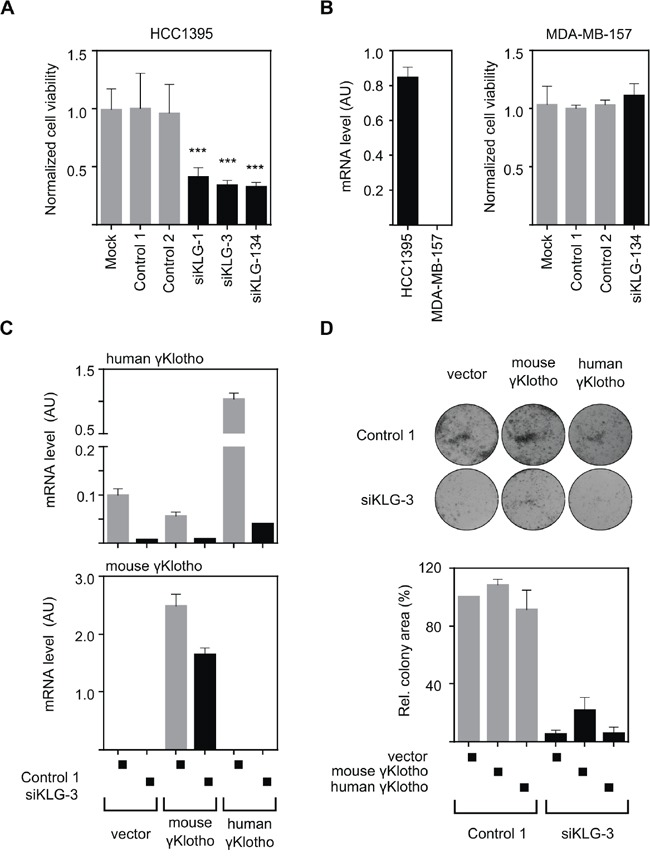
The growth inhibitory effect of γKlotho knockdown is specific **A.** Two individual γKlotho siRNAs (siKLG-1 and siKLG-3) decrease HCC1395 cells viability to a similar extent as the siRNA pool (siKLG-134). Cell viability was determined by MTS assay 5 days after transfection. Means ± SD of 4 wells are shown. *** indicates *p* ≤ 0.001. **B.** γKlotho negative MDA-MB-157 cells (as assessed by qRT-PCR-left pannel) are not affected by γKlotho siRNA. Cell viability was assessed as in panel A. **C.** Mouse γKlotho is partially resistant to siKLG-3. HCC1395 cells stably overexpressing mouse or human γKlotho-Flag protein were transfected with siRNA and mRNA levels for mouse and human γKlotho determined by qRT-PCR. Data represent mean of three replicates ± SD. **D.** The siRNA-resistant mouse γKlotho gene in part rescues HCC1395 cells after γKlotho knockdown. HCC1395 cells stably overexpressing mouse or human γKlotho-Flag proteins were transfected with siKLG-3 against human γKlotho, 2 days later plated in 12-well plate (5000 cells/well) and cultured for 10 days. Representative images of colony formations are shown. Cell growth was quantified by determining the percentage of area covered by crystal violet stained cell colonies using ImageJ ColonyArea plugin and is presented relative to control. Data are presented as mean of three independent experiments ± SD.

In order to ensure that the observed growth-inhibitory phenotype after γKlotho knockdown was specific, we first examined if individual siRNAs gave the same phenotype as the siRNA pool. Two different siRNAs with comparable silencing efficiencies, siKLG-1 and siKLG-3 (Supplementary Figure S3), induced similar viability inhibition as the siRNA pool (Figure 4A). Importantly, the viability of γKlotho-negative cell line MDA-MB-157 was not altered by siRNA treatment targeting γKlotho (Figure 4B). Next, we aimed to rescue the γKlotho siRNA growth inhibitory phenotype by the expression of a mouse gene that is relatively resistant to siKLG-3. First, we established HCC1395 cell lines stably overexpressing the human or mouse γKlotho-Flag gene (Figure 4C, top panel). siKLG-3 caused significant reduction in human γKlotho mRNA levels (>90%), while the expression of the mouse gene was decreased only by 30% (Figure 4C, bottom panel). Knockdown of γKlotho significantly reduced colony formation of vector transfected and human γKlotho-Flag expressing cells, whereas the expression of the mouse γKlotho-Flag partially complemented the ability of cells to form colonies in the presence of siKLG-3 (Figure 4D). In these HCC1395 stable cells, where endogenous human γKlotho is already expressed at high levels, further overexpression does not significantly alter cell viability or growth, suggesting a threshold level of required γKlotho protein is already present. As a whole, these results confirmed that the growth inhibitory effect of γKlotho knockdown is specific and further suggest dependence of a subset of triple negative breast cancer cells on γKlotho expression.

### γKlotho knockdown leads to cell cycle arrest and apoptosis

**Figure 5:**
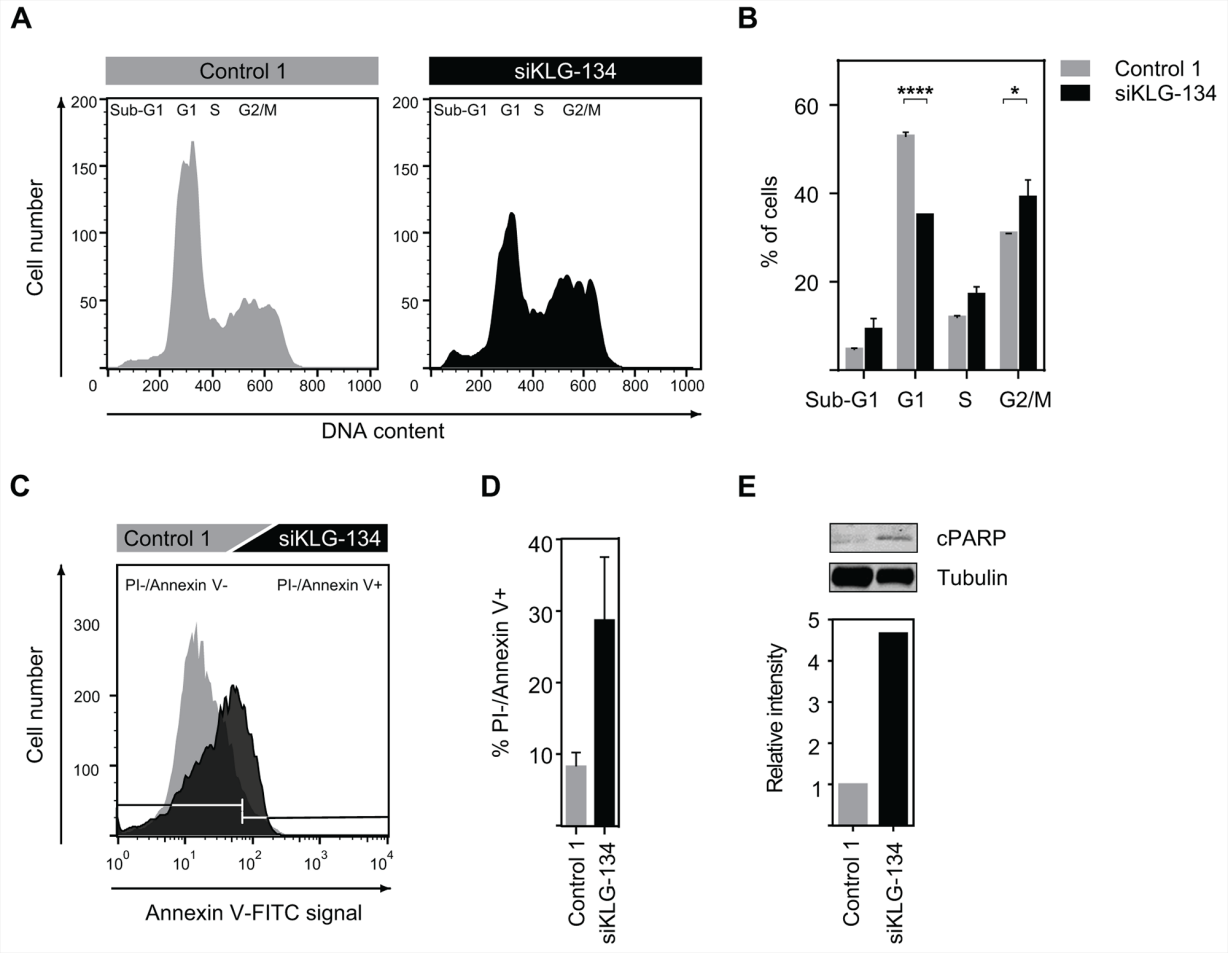
γKlotho knockdown leads to cell-cycle arrest and apoptosis **A.** siRNA knockdown of γKlotho arrests cells in cell cycle. HCC1395 cells were transfected with a siRNA pool against γKlotho (siKLG-134) and corresponding controls. 2 days after transfection cells were seeded in 6-well plates and harvested 3 days later. Equal numbers of control and siRNA-treated cells were fixed, stained with PI, and analyzed for DNA content. Representative flow cytometry histograms are shown. **B.** The percent of cells in different phases of cell cycle. Data are presented as mean ± SD of 2 replicates. **p ≤ 0.01, ****p ≤ 0.0001; one-way ANOVA. **C.** γKlotho knockdown leads to apoptosis of HCC1395 cells. Cells were transfected with indicated siRNAs, 2 days after transfection seeded in 6-well plates, and harvested 3 days later. Cells were stained for Annexin V and PI and analyzed by FACS. Representative histograms are shown. **D.** Quantification of PI-/Annexin+ cells. Data are mean ± SD of two biological replicates. **E.** Cells were transfected with indicated siRNAs, 2 days after transfection cells seeded in 6 well plates, grown for 3 days, serum-starved overnight, harvested and immunobloted for cleaved PARP-1 and tubulin. Representative immunoblot of two experiments is shown. Immunoblots were quantified by Li-COR Odyssey Imager.

We have shown that knockdown of γKlotho in HCC1395 and MDA-MB-231 cells results in reduced cell viability and diminished clonogenic growth (Figure 3). To further determine the molecular function of γKlotho in TNBC cells we analyzed the impact of γKlotho ablation on cell cycle progression and apoptosis. HCC1395 cells were transfected with control and γKlotho siRNA and analyzed by flow cytometry 5 days after transfection. γKlotho knockdown resulted in significant reduction of cells in G1 phase and concomitant accumulation in S and G2/M phases of the cell cycle (Figure 5A and 5B) compared to control siRNA transfected cells, suggesting a blockade in cell cycle progression through S/G2 phases. In addition, we observed an increase in the subG1 peak indicating increased apoptosis (Figure 5A and 5B). To further characterize the effects of γKlotho loss we used Annexin V/propidium iodide staining. γKlotho siRNA treated cells underwent apoptotic cell death as evidenced by positive Annexin V staining (Figure 5C and 5D). Consistent with this, we detected an increase in the cleaved form of PARP-1 by Western blot analysis (Figure 5E), indicative of apoptosis. Altogether, these results further confirm that in γKlotho positive TNBC cells depletion of γKlotho leads to cell cycle arrest and apoptotic cell death consistent with oncogenic properties for this gene.

### Constitutive ERK activation induced by γKlotho knockdown

**Figure 6:**
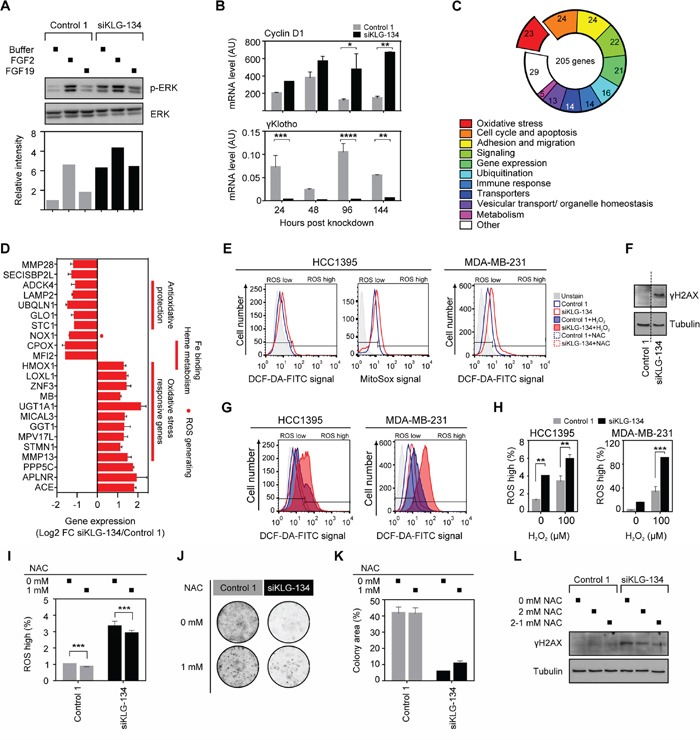
γKlotho depletion increases oxidative stress **A.** Increased ERK phosphorylation level after γKlotho knockdown, but the response to growth factor FGF2 is blunted; FGF19 has no effect on ERK phosphorylation in HCC1395 cells. Cells were transfected with indicated siRNAs, 2 days after transfection seeded in 6-well plates, grown for 3 days, serum-starved overnight, and treated for 10 min with FGF2/19 or buffer. Cell lysates were immunoblotted for phospho- and total ERK. Immunoblots were quantified by Li-COR Odyssey Imager. **B.** The bona fide ERK target gene cyclin D1 expression is increased after γKlotho knockdown. HCC1395 cells were transfected with indicated siRNAs, harvested after 1–4 days and gene expression measured by qRT-PCR. Data were normalized against Rplp0 and are presented as mean ± SD of 2 replicates. *p ≤ 0.05, **p ≤ 0.01; two-way ANOVA. **C.** Gene expression signature after γKlotho knockdown. HCC1395 cells were transfected with siRNA pool against γKlotho and corresponding control, harvested after 2 days and RNA analyzed by the Illumina microarray. Genes that showed log2 fold-change of > 1 and *p* < 0.05 were considered to be differentially expressed. Using NCBI resources (Pubmed, Entrez, Unigene) the gene list was manually categorized by gene functions integrated into the well-defined pathways and functional groups important for carcinogenesis. The number of genes in each differentially expressed category is shown in the pie chart. **D.** Genes involved in the oxidative stress homeostasis and oxidative stress responsive genes represent one of the most prominent differentially regulated functional groups after γKlotho knockdown. **E.** γKlotho knockdown causes an increase in ROS levels. HCC1395 and MDA-MB-231 cells were transfected with indicated siRNAs, 2 days after transfection seeded in 6 well plates, and harvested 3 days later. Before harvest, 10 μM DCF-DA/5 μM MitoSox was added to the cells for 45/15 minutes at 37°C, cells washed with PBS, trypsinized, and stained with PI before FACS analysis. Representative histograms are shown. **F.** MDA-MB-231 cells were transfected with indicated siRNAs, 2 days after transfection cells were seeded in 6-well plates, grown for 3 days, harvested and immunobloted for γH2AX and tubulin. Representative immunoblot of two experiments are shown. Vertical dotted line marks place in the gel where unrelated lanes were removed for clarity of presentation. **G.** γKlotho siRNA transfected cells were treated with 100 μM H_2_O_2_ for 15 min, DCF-DA staining was followed by FACS analysis as in E. **H.** Quantification of PI-/ROS+ cells. Data are mean ± SD of 2 biological replicates. **p ≤ 0.01, ***p ≤ 0.001; two-way ANOVA. **I.** HCC1395 cells were transfected with indicated siRNAs, and from the following day maintained in the presence or absence of 1 mM NAC. Cells were seeded in 6 well plates 3 days after transfection and harvested 2 days later. MitoSox staining was followed by FACS analysis as in E. **J.** HCC1395 cells were transfected with indicated siRNAs, and from the following day maintained in the presence or absence of 1 mM NAC. 2 days after transfection, cells were seeded in 24-well plates (1000 cells/well) and cultured for 11 days, then colonies were visualized. **K.** Cell growth was quantified by determining the percentage of area covered by crystal violet stained cell colonies using ImageJ ColonyArea plugin. Data are presented as mean ± SD. **L.** Cells were transfected with indicated siRNAs, 2 days after transfection cells seeded in 6-well plates, grown for 3 days in the absence or presence of 2 mM NAC (2-1 mM NAC media changed with 1 mM for last 2 hours before harvest), harvested and immunobloted for γH2AX and tubulin. Representative immunoblot of two experiments are shown.

We have previously shown that γKlotho can bind to FGFRs [24]. To determine the role of γKlotho in FGF signaling in TNBC cells, we treated HCC1395 cells with the canonical FGF, FGF2, or an endocrine FGF, FGF19, after mock or γKlotho knockdown and overnight serum starvation (Figure 6A). Unexpectedly, when γKlotho was knocked down we observed a significant increase in ERK activation in the absence of any added FGF ligand (Figure 6A). FGF2 induced FGFR signaling as detected by a strong ERK phosphorylation, however, the fold induction was decreased in γKlotho siRNA treated cells, likely due to the higher basal phosphorylation levels (Figure 6A). In contrast to FGF2, we did not observe activation of the ERK pathway by FGF19 although all FGFRs are expressed in HCC1395 cells (Supplementary Figure S4A). These results indicate that in γKlotho positive TNBC cells, depletion of γKlotho causes persistent activation of the ERK signaling pathway, also shown by increases in a direct target of ERK signaling [38] (Figure 6B). Forced expression of Klotho was previously shown to inhibit MCF7 breast cancer cell growth by inhibiting insulin/IGF-1/AKT signaling [30]. To determine whether γKlotho also interferes with this signaling pathway, MDA-MB-231 cells overexpressing γKlotho were treated with insulin and AKT phosphorylation was measured by Western blot analysis (Supplementary Figure S4B). In contrast to Klotho, γKlotho does not interfere with AKT activation by insulin/IGF-1.

### γKlotho is critical for maintaining ROS balance in cancer cells

To begin to understand the cellular mechanisms that may contribute to the sensitivity of TNBC cells to γKlotho depletion, we performed microarray gene expression analysis of HCC1395 cells harvested 2 days after treatment with γKlotho siRNA or the corresponding control (*n* = 2). At this time point γKlotho mRNA expression was reduced by 90% (Supplementary Figure S4C). 205 genes showed significant differential expression having log2 fold-change of > 1 and *p* < 0.05. Of these, at least 64 were previously shown to be involved in cancer pathogenesis (Supplementary Figure S4D). Indeed, 68% of identified oncogenes were down-regulated and 70% of identified tumor-suppressors were upregulated upon γKlotho knockdown (Supplementary Figure S4D). Among differentially regulated genes, genes functioning in cell cycle/apoptosis, cell adhesion/migration and signaling pathways were well represented (Figure 6C). Intriguingly, one of the most prevalent altered functional groups were genes known to be regulated by oxidative stress, involved in generating or protecting against reactive oxygen species (ROS) (Figure 6C and 6D). Several genes that are critical for the protection against oxidative damage, including STC1, GLO1, UBQLN11 and LAMP2 [39–44] were repressed in γKlotho siRNA treated cells. In contrast, several genes that are typically activated by oxidative stress, such as HMOX-1, GGT1, UGT1A1 and MMP13 were significantly induced in γKlotho knocked down cells (Figure 6D), suggesting elevated ROS levels in cells depleted of γKlotho. To determine if ROS levels were increased in γKlotho depleted cells, we performed flow cytometry analysis of knocked down or control cells incubated with DCF-DA or Mitosox. As hypothesized, we detected increased levels of ROS in γKlotho depleted HCC1395 and MDA-MB-231 cells (Figure 6E and 6H). Concomitant with increased ROS, we observed the accumulation of DNA damage marker γH2AX in γKlotho depleted cells (Figure 6F). Furthermore, γKlotho depleted cells were less resistant to additional oxidative stress induced by H_2_O_2_ as shown by higher ROS levels upon treatment (Figure 6G and 6H). On the other hand, treatment of γKlotho siRNA treated cells with antioxidant N-acetylcysteine (NAC) caused a reduction in ROS levels (Figure 6I), partly rescued cell viability (Figure 6J and 6K) and reduced oxidative damage as shown by lower levels of γH2AX (Figure 6L). Our results suggest γKlotho confers resistance to the oxidative stress commonly found in cancer cells.

**Figure 7:**
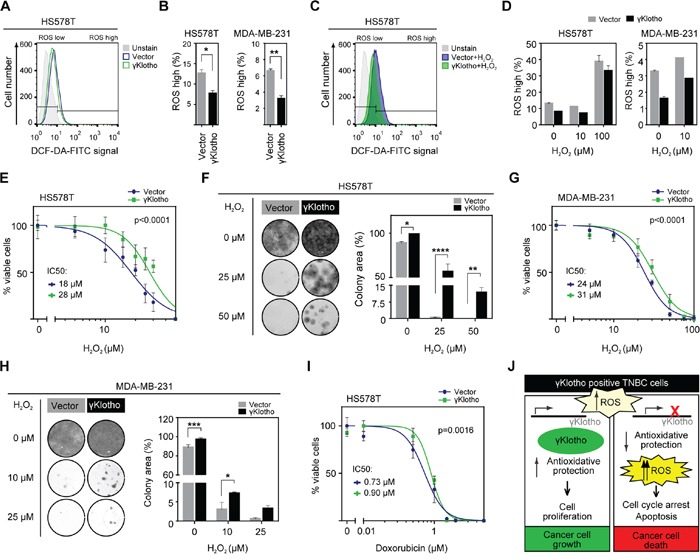
γKlotho protects cancer cells against ROS toxicity **A.** γKlotho overexpression reduces ROS levels in unstressed cells. Cells overexpressing control vector or γKlotho-Flag were seeded in 6 well plates, and harvested the next day. Before harvest, 10 μM DCF-DA was added to the cells, cells were incubated for 45 minutes at 37°C, then washed with PBS, trypsinized, and stained with PI before FACS analysis. Representative histograms are shown. **B.** Quantification of ROS positive live HS578T and MDA-MB-231 cells overexpressing control vector or γKlotho-Flag. Data are mean ± SD of 2 replicates. *p* ≤ 0.05, **p ≤ 0.01; *t*-test **C.** Control vector or γKlotho-Flag overexpressing cells were treated with 10 μM or 100 μM H_2_O_2_ when 10 μM DCF-DA was added to the cells. Staining and FACS analysis was performed as in A. Representative histograms are shown. **D.** Quantification of ROS positive live cells. Data are mean ± SD of 2 replicates. **E.** HS578T cells stably expressing vector or γKlotho-Flag were seeded in 96-well plates at 6500 cells/well. 12 hours later, culture media was replaced with increasing concentration of H_2_O_2_ in serum free RPMI media. 72 hours later MTS viability assay was performed. Data are normalized to the untreated controls (100% viability). Dose response curves are plotted using a non-linear regression model and IC_50_s were determined from the fitted curves using Hill equation. **F.** 100,000 HS578T cells expressing control vector or γKlotho were seeded in 12-well plates, next day treated with the indicated concentrations of H_2_O_2_ in serum free RPMI for 1 h at 37°C and then re-plated into 12-well plates at low density (2500 cells/well). Two weeks after plating, cells were fixed in ice-cold methanol and stained with crystal violet. Representative images of 2 independent experiments are shown. Cell growth was quantified by determining the percentage of area covered by crystal violet stained cell colonies. Data are presented as mean of three replicates ± SD from one of the experiments. *p ≤ 0.05, **p ≤ 0.01, ****p ≤ 0.0001; two way ANOVA. **G.** MDA-MB-231 cells stably expressing control vector or γKlotho-FLAG were seeded in 96-well plates at 8000 cells/well and cell viability after H_2_0_2_-induced oxidative stress measured as in E. **H.** 100,000 MDA-MB-231 cells expressing control vector or γKlotho were treated as in F, then re-plated in 12-well plates at 5000 cells/well. Cell growth was quantified and is presented as in F. *p ≤ 0.05, ***p ≤ 0.001; two way ANOVA. **I.** HS578T-vector and γKlotho-Flag cells were treated with increasing concentration of doxorubicin as in E. **J.** The working model of γKlotho role in triple negative breast cancer cells.

To further confirm the role of γKlotho in protection against high ROS levels, we measured ROS levels in MDA-MB-231 and HS578T cells overexpressing γKlotho. As expected, ROS levels were lower in unstressed (Figure 7A and 7B) and H_2_O_2_-stressed cells (Figure 7C and 7D) overexpressing γKlotho. Importantly, γKlotho expression provided protection against toxicity induced by H_2_O_2_ or doxorubicin (Figures 7E–7I and S5) as measured by both MTS viability assays and liquid colony formation upon acute oxidative stress. Furthermore, γKlotho provided additional protection to NAC pretreated cells (Supplementary Figure S5). Together, our results provide strong evidence that γKlotho contributes to maintaining reactive oxygen homeostasis thus enabling TNBC cells to thrive in high ROS conditions.

## DISCUSSION

To date, no targeted therapy exists for the treatment of TNBC. Heterogeneity of TNBCs and lack of common drugable molecules/pathways have been major roadblocks in the development of efficient therapies against this breast cancer subtype [4]. There is a need to identify common molecular targets and biomarkers that can be used for the development of effective treatment strategies for patient subgroups [45]. A few pathways have been recently suggested as potential therapeutic targets in specific subsets of TNBCs, such as IGF-1R/FAK, WNT and VEGF [46–48]. Here, we discovered that γKlotho, an uncharacterized member of the Klotho family, is significantly upregulated in triple negative breast cancer (Figure 1). γKlotho expression positively correlated with higher Ki67 index (Table 1). Consistent with this, high γKlotho-expressing patients harbored higher stage and grade tumors and had worse progression-free survival (Figure 1C and Supplementary Table S2). In agreement with our findings, γKlotho was previously shown to be required for the growth of established, transformed cells [49] in colon tumorigenesis.

γKlotho was overexpressed in more than 60% of all TNBC samples in our study (13 of 19 TN samples with fold change > 1.4). In the Curtis and TCGA datasets, 27–41% of TN samples highly overexpressed γKlotho compared to the average tumor expression (Supplementary Table S3). In addition to p53 loss found in 80% of TN breast tumors, and PIK3CA mutations, occurring in 11% of cases, several genes, such as EGFR, AR, FGFR have been found to be overexpressed in a significant portion of TNBC [50–52]. Some of these genes are being used for the development of targeted therapies, although the success rate is still low, potentially due to diverse compensatory mechanisms. Our analysis uncovered γKlotho as a novel marker for a substantial portion of TNBC patients that correlates with worse progression of the disease. Given its restricted expression profile in normal tissues [24], γKlotho may represent very specific potential target for treatment of TNBC.

Our finding of γKlotho overexpression in cancer was unexpected, because the other two members of the Klotho family, Klotho and βKlotho, are downregulated in several cancers. Klotho is an established tumor suppressor, epigenetically silenced in breast and other cancers [31, 32, 53–55] since it inhibits signaling pathways, such as IGF-1, PI3K, WNT, and TGFβ [30, 56–58]. βKlotho was similarly shown to suppress tumor growth in hepatocellular carcinoma [33, 34]. The three Klothos belong to the glycosidase family 1 and share structural similarities to β-glycosidases. The critical residues required for enzymatic activity are not conserved in any of the Klotho proteins [59] implying their biological function is independent of this glycosidase activity. Interestingly, unlike Klotho and βKlotho, γKlotho contains only one β-glycosidase-like domain in the extracellular region (Supplementary Figure S1A), potentially differentiating its function. In this study we found that in TNBC γKlotho has the opposite expression and function than the other two Klotho members. Furthermore, by analyzing TCGA datasets we also found γKlotho overexpressed in other cancer types (Supplementary Figure S1E), suggesting γKlotho oncogenic activity is not restricted to breast cancer and might be linked to the specific oxidative tumor microenvironment or metabolic state of particular cancer types.

Our study shows that γKlotho is not solely a marker for a subset of TNBC but also promotes cancer cell growth (Figure 2) and is necessary for the survival of TN breast cancer cells expressing this gene (Figure 3). Both gain of function and loss of function experiments demonstrated that TNBC cells expressing γKlotho are dependent on its presence for viability and clonogenic growth and that this gene confers a growth advantage. Indeed, depletion caused cell cycle arrest and apoptotic death (Figure 5).

γKlotho belongs to a family of coreceptors for endocrine FGFs [21–23] that can also modulate signaling of canonical ligands [60]. Since γKlotho can bind FGFRs [24], we evaluated the role of FGF signaling in γKlotho-mediated regulation of cell viability. Surprisingly, we found activated ERK even in the absence of any FGF stimulation in γKlotho depleted cells (Figure 6A). This result suggested increased oxidative stress in these cells since ERK activation is normally linked to proliferation and growth, but its prolonged activation is observed under high ROS and is detrimental to cells [61, 62]. We found increased ROS levels and induction of oxidative stress genes after γKlotho knockdown followed by apoptotic death (Figure 6). Furthermore, we show that forced expression of γKlotho confers resistance to oxidative stress (Figure 7). Thus, we propose that in TNBC cells, and perhaps also in other cancers (Supplementary Figure S1E) γKlotho is involved in protection of tumor cells against increased oxidative stress and thus confers a survival advantage. Although Klotho has been shown to regulate endogenous oxidative balance [63], it remained undetectable in γKlotho knockdown cells. Klotho knockout mice have increased oxidative stress that underlies accelerated aging, and Klotho overexpression has a protective effect [64–68]. Intriguingly, our data suggest that although Klotho and γKlotho seem to both provide protection against oxidative stress, they have opposite roles in cancer. This may be due to the regulation of distinct pathways in ROS homeostasis. Several pathways have been already proposed for Klotho such as inhibition of IGF-1 signaling [64–65, 68]. We show that γKlotho does not interfere with IGF signaling (Supplementary Figure S7B), further distinguishing it from Klotho at the functional level. Instead, it appears that γKlotho provides increased protection against high ROS, as several antioxidant genes were downregulated after γKlotho knockdown, most of which are not known to be regulated by Klotho (Figure 6D). These include glyoxalase 1 (GLO1), an enzyme that reduces glycative and oxidative stress [69], and several genes involved in heme metabolism and iron homeostasis. Triple negative tumors have been shown to have high levels of GLO1 indicative of this tumor's requirement for antioxidant protection [70]. We found that γKlotho confers protection against basal and H_2_O_2_/doxorubicin induced oxidative stress (Figure 7), suggesting this gene is part of an anti-oxidant network in TN tumors. Thus, we propose that γKlotho gives TNBC cells an advantage to survive under high ROS conditions (Figure 7J).

In summary, our study defines γKlotho as a novel marker and potential oncogene for a subgroup of TNBCs, necessary for protecting rapidly growing cells against increased oxidative damage. γKlotho may therefore be a potential novel therapeutic target or a biomarker for TNBC or other tumors with significant oxidative stress or low antioxidant defense which may be susceptible to ROS causing therapies.

## MATERIALS AND METHODS

### Patients and clinical samples

Sixty-eight frozen samples of breast cancer tissue and surrounding normal (benign) breast tissue (68 paired samples) were obtained from the Tissue Procurement Core at UT Southwestern Medical Center, Dallas under Institutional Review Board approval. Samples covered all the pathological disease stages and histological subtypes and were gained from patients diagnosed for breast cancer in the period between 2001 and 2008. Patient clinical information (including race, age at diagnosis, clinical stage, pre- and post-surgical therapy) and histopathology reports showing histological subtype, tumor grade and molecular status (ER, PR, HER2) were available for all the specimens. Expression patterns of ER, PR, HER2 receptors and Ki67 proliferation index were assessed by immunohistochemistry and were, in addition to tumor grade, used for classifying tumors into molecular subtypes of breast cancer. Tumors were classified as luminal A – LumA (tumor grade 1 or 2 and ER+/PR ±/HER2-, Ki67 ≤ 14%, *N* = 13), luminal B – LumB (higher tumor grade and ER+/PR ±/HER2-, Ki67 > 14% or ER+/PR ±/HER2+, *N* = 30), HER2 overexpressing – Her2 (ER-/PR-/HER2+, *N* = 6) and triple negative breast cancer subtype - TNBC (ER-/PR-/HER2-, *N* = 19. The Ki67 cut-off point was determined according to Cheang et al. [71].

### Cell culture and transfections

Human breast cancer cell lines (gift from Dr. David Euhus or purchased from ATCC) HCC1954, HCC1428, MCF7, BT474, T47D, HCC1419, MDA-MB-231, MDA-MB-157, HCC38, HCC1395, HCC1806, HS578T and BT20 were maintained in RPMI-1640 medium (Gibco™ Life Technologies) supplemented with 10% FBS (Invitrogen). siRNA transfections were performed with Lipofectamine^®^ RNAiMAX (Thermo Fisher Scientific) according to the manufacture's protocol. siRNA duplexes targeting γKlotho were purchased from Sigma-Aldrich: siKLG-1, 5′-GUGAUGAGUGGAGAAUUCA-3′; siKLG-2, 5′-GAUGUAGCCUGUGACGGCU-3′; siKLG-3, 5′-CUAUCCAAAGGCUUCAGUU-3′; siKLG-4, 5′-GUUGGUACCUCAAAGCUUU-3′. A non-targeting control siRevL1 (5′-ACUACAUCGUGAUUCAAACUU-3′), further referred as Control 1, was purchased from ThermoScientific. SiLonRF pool, a siRNA control that targets an endogenous gene irrelevant to the gene of interest, was purchased from Dharmacon (Control 2: 5′-GCACUGCCGACAUUGAAUA-3′, 5′UCACACAGCUGUUGGAAG A-3′, 5′GACCAAGAAUGUUCCAAUA-3′, and 5′UCAGAGAGCUUCAUGAUUU-3′). Expression vectors for full-length mouse γKlotho, human γKlotho and empty vector were introduced into HCC1395, HS578T, and MDA-MB-231 cells using X-tremeGENE HP Transfection Reagent (Roche) or Lipofectamine^®^ 2000 Transfection Reagent (Thermo Fisher Scientific) following manufacturer's instructions. Stable clones were selected with 700 μg/ml of G-418 (Roche) and maintained at 400 μg/ml of G-418.

### RNA preparation, cDNA synthesis, and quantitative reverse transcription PCR analysis (qRT-PCR)

RNA was extracted using RNAStat60 (TelTest) according to the manufacturer's directions. Genomic DNA contamination was eliminated by DNase I (Roche) treatment in 4.5 mM MgCl_2_. cDNA for qPCR assays was prepared from 4 μg (tissue samples) or 1 μg (cell lines) DNase-treated RNA using High Capacity cDNA Reverse Transcription kit (Life Technologies). Gene expression levels were measured in triplicate wells of a 384-well reaction plate with 10 ng cDNA per well on an Applied Biosystems 7900HT with SYBR Green chemistry using the following primers: human Klotho forward, 5′-TCAAAAAGTTCATCATGGAAACC-3′ and reverse, 5′-ATGAGGGACCATGCGGTAT-3′; human βKlotho forward, 5′-ACCACGGCCATCTACATGAT-3′ and reverse, 5′-CGTATTTCATCTAACCTTATTGCTTG-3′; human γKlotho forward, 5′-GCAGCACACCACATCATTAAGG-3′ and reverse, 5′-CTGCGCCACGTGGTGTTA-3′; mouse γKlotho forward, 5′-AAGTTACATTGCACTCAGTTCTGC-3′ and reverse, 5′-CCCTTTTATATCCACGCCATC-3′; human FGFR1 forward, 5′-AGGCTACAAGGTCCGTTATGC-3′ and reverse, 5′-TGCCGTACTCATTCTCCACAA-3′; human FGFR2 forward, 5′-TTAAGCAGGAGCATCGCATTG-3′ and reverse, 5′-GGGACCACACTTTCCATAATGAG-3′; human FGFR3 forward, 5′-CCTCGGGAGATGACGAAGAC-3′ and reverse, 5′-CGGGCCGTGTCCAGTAAGG-3′; human FGFR4 forward, 5′-TGCAGAATCTCACCTTGATTACA-3′ and reverse, 5′-GGGGTAACTGTGCCTATTCG-3′; human cyclophilin B forward, 5′-GGAGATGGCACAGGAGGAA-3′ and reverse, 5′-GCCCGTAGTGCTTCAGTTT-3′; human Rplp0 forward, 5′-CGAGGGCACCTGGAAAAC-3′ and reverse, 5′-CACATTCCCCCGGATATG-3′. Primers were designed using Roche Universal Probe Library Assay Design Center and used in 150 nM concentration. For primer validation and efficiency determination, standard human/mouse universal cDNA was used. PCR efficiencies were calculated from the slope of the resulting standard curves. qRT-PCR data were analyzed by ABI instrument software SDS2.1. Baseline values of amplification plots were set automatically and threshold values were kept constant to obtain normalized cycle time and linear regression data. Normalized mRNA levels, which are expressed as arbitrary units (AU), were obtained by dividing the averaged, efficiency corrected values for mRNA expression by that for reference gene (cyclophilin B and/or Rplp0) [72]. The resulting values were multiplied by 10^2^ for graphical representation unless otherwise stated.

### Antibodies and plasmid constructs

Antibodies used in this study were as follows: anti-ERK (Cell Signaling Technology # 9102), anti-P-ERK (Cell Signaling Technology # 4370), anti-Tubulin (Sigma Aldrich #T6199), and anti-cleaved PARP (Cell Signaling Technology#9541), anti-P-AKT (S473, Cell Signaling Technology#4060P), anti-AKT (Cell Signaling Technology#4961P), anti-γH2AX (Milipore# 07-164). cDNAs containing the mouse or human γKlotho were cloned into the pEF1 vector (Invitrogen) with a Flag-epitope tag at the C terminus.

### Cell viability assays, colony formation, and anchorage-independent growth soft agar assays

Cell viability was assessed by standard MTS assays using Cell Titer reagents (Promega, Madison, WI, USA) according to the manufacturer's protocols. Cells were transfected with siRNAs, 48 hours later re-plated at 2000 cells (1000 cells for HS578T) per well in 96-well plates and their viability assessed over 4-day period or in a single end point, on day 5 or day 6 post-transfection. Each condition contained 6 replicates unless mentioned otherwise. Viability of plasmid-transfected cells was determined in the same manner. Absorbance at 490 and 650 nm (reference wavelength) was measured by a Spectra Max (Molecular Devices, Sunnyvale, CA, USA) or a FlouroStar Omega (BMG Biosciences, Ortenberg, Germany) plate reader. For colony formation assays on plastic, 48 hours after siRNA transfection cells were re-plated into 12-well plates at low density. HS578T and MDA-MB-231 cells stably expressing vector or γKlotho-Flag were also plated into 12-well plates at low density. Approximately two weeks after plating, cells were fixed in ice-cold methanol and stained with crystal violet (0.5% (w/v) in 25% methanol) for 20 minutes, extensively washed in dH_2_O and then counted. Quantification of the results was performed with Image J (ColonyArea plugin). For anchorage-independent soft agar growth assays, 48 hours after siRNA transfection 1–5 × 10^4^ cells were suspended in medium containing 0.35% Noble agar (Difco) and overlaid on 0.5% Nobel agar in triplicate in 6-well plates. Cells were allowed to grow for 3–4 weeks before colonies were quantified. ImageJ software was used for robust, unbiased quantification of colony size and number using 33 pictures taken for each experimental condition.

### Cell survival analysis upon oxidative stress

The cytotoxic effect of H_2_O_2_ and doxorubicin was tested in MDA-MB-231/HS578T cells stably expressing vector and γKlotho-Flag using the MTS viability assay. Exponentially growing cells were seeded in 96-well plates at 8000/6500 cells/well. 12 hours later, culture media was replaced with increasing concentration of H_2_O_2_ and doxorubicin in serum free RPMI media. 48–72 hours later MTS viability assay was performed. Each cell line was tested in 2–4 independent assays with 3–6 replicates per condition. Data were normalized to the untreated controls (100% viability). Dose response curves were plotted using a non-linear regression model and IC_50_s were determined from the fitted curves. Data were fitted to the Hill equation, y=Top/(1+10[(log(EC_50_/x)) × HillSlope]) using GraphPad Prism.

To further examine resistance of vector and γKlotho expressing MDA-MB-231 and HS578T cells to oxidative stress, equal number of cells (100,000/well) were seeded in 12-well plates, next day treated with indicated concentrations of H_2_O_2_ in serum free RPMI for 1 h at 37°C and then re-plated into 12-well plates at low density (1000, 5000 cells/well). Two weeks after plating, cells were fixed in ice-cold methanol and stained with crystal violet.

### Cell cycle analysis, Annexin V apoptosis assay, and ROS measurements

For cell cycle analysis, apoptosis assay and ROS measurements, cells were transfected with siRNAs, 2 days later re-plated into 6-well plates at density 1.5 × 10^5^ cells per well and harvested after 3 days. For cell cycle analysis, 1 × 10^6^ cells were fixed in ice-cold 70% ethanol over night at −20°C, washed twice with PBS and stained with a solution containing 20 μg/ml PI and 225 μg/ml RNase at room temperature for 30 min. For apoptosis evaluation, cells were harvested, washed with ice-cold PBS, suspended in binding buffer and incubated with Annexin V-FITC antibody and PI according to the manufacturer's instructions (BD Pharmingen). Intracellular ROS generation was measured by staining the cells with 10 μM DCF-DA (Thermo Fisher Scientific) for 45 min at 37°C or with 5 μM MitoSox (Thermo Fisher Scientific) for 15 minutes at 37°C. When indicated, cells were pretreated with 1–2 mM NAC for 24 or 48 hours or treated by 10/100 μM H_2_O_2_ for 15 minutes prior to (HCC1935 and MDA-MB-231 after knockdown) or after addition of DCF-DA (HS578T and MDA-MB-231 stable cells). After staining, cells were washed with PBS and collected for flow cytometer analysis. All samples were analyzed by a BD FACSCalibur and data analyzed using FlowJo software.

### Western blots

Cells were lysed in RIPA buffer (50 mM Tris, 150 mM NaCl, 0.1% SDS (w/v), 0.5% sodium deoxycholate (w/v), 1% Nonidet P-40 (v/v)), with protease and phosphatase inhibitors (Sigma). Lysates were clarified by centrifugation at max speed for 10 minutes at 4°C. Lysates were then resolved by SDS-PAGE, transferred to nitrocellulose membrane (Bio-Rad), and the protein blots incubated with specific antibodies (1:1000). Signal was visualized using IRDye^®^ secondary antibodies (1:15000) and the Odyssey Imager (LI-COR) was used for detection and quantification.

### Microarray gene expression analysis

Cells were treated with siRNAs and 2 days later RNA was extracted using the RNAStat60 (TelTest) and purified by the RNeasy kit (Qiagen). RNA quality was evaluated with the Experion gel system (BioRad), prior to labeling at UTSW's microarray core. Labelled RNA was hybridized to Illumina human HT-12 version 4 microarray chips according to the manufacture's protocol. Microarray analysis was done with in-house Visual Basic software MATRIX 1.508, a gift of Dr. Luc Girard. Array data were quantile-normalized and samples were compared by calculating log2 ratios for each gene along with a *t*-test *P*-value. Oncogenes or tumor suppressor genes that showed log2 fold-change of > 1 and *p* value < 0.05 are reported in Supplementary Figure S4. The gene list was manually categorized by gene function using NCBI resources (Entrez, Pubmed, etc.). GEO accession number is GSE69082.

### Statistical data analysis

RNA-Seq and clinical data of breast invasive carcinoma and other cancers were downloaded from The Cancer Genome Atlas (TCGA) data portal (https://tcga-data.nci.nih.gov/tcga) on June 14th, 2014 and tested for associations as indicated [73]. Gene expression levels for Klotho, βKlotho and γKlotho were estimated by the RNA-Seq Expectation-Maximization (RSEM) normalization method. The median of each gene was calculated for all available patients. Tumors with negative estrogen receptor (ER) status, progesterone receptor (PR) status, and HER2 status by either IHC or FISH were considered as triple negatives. Tumors with undetermined or not evaluated status for any one of these parameters were excluded. Progression-free survival was calculated from the date of breast cancer diagnosis to the date of detection of distant metastasis or the date of death, whichever was earlier. Overall survival was calculated from the date of diagnosis to the date on which the patient died from any cause. Patients alive at the end of the study period were censored at the date of last follow-up or the last date the patient was known to be alive, whichever was later. Kaplan-Meier survival curves and log-rank tests were calculated with SPSS Statistics 17. For the Curtis microarray dataset, the expression of Klotho genes was similarly analyzed as above in about 2,000 samples (*n* = 144 benign, 320 TN, 1673 nonTN), with Bonferroni post-hoc test.

Other statistical analysis were performed using GraphPad Prism, version 6 (GraphPad Softwar, CA, USA). *P*-values were calculated using (paired) Student's *t*-tests, one-way or two-way ANOVA with Bonferroni post-hoc tests. Correlation between two ranked variables was assessed using a nonparametric Spearman rank correlation test. *P*-values < 0.05 were considered as statistically significant. Values that were not normally distributed were log-transformed in order to meet the assumptions of parametric statistical tests.

## SUPPLEMENTARY FIGURES AND TABLES


