# Correction: γKlotho is a novel marker and cell survival factor in a subset of triple negative breast cancers

**DOI:** 10.18632/oncotarget.26649

**Published:** 2019-01-25

**Authors:** Nuša Trošt, Samuel Peña-Llopis, Sajjan Koirala, Jurij Stojan, Patrick Ryan Potts, Klementina Fon Tacer, Elisabeth D. Martinez

**Affiliations:** ^1^ Institute of Biochemistry, Faculty of Medicine, University of Ljubljana, Ljubljana, Slovenia; ^2^ Department of Pharmacology, University of Texas Southwestern Medical Center, Dallas, Texas, USA; ^3^ Department of Physiology, University of Texas Southwestern Medical Center, Dallas, Texas, USA; ^4^ Hamon Center for Therapeutic Oncology Research, University of Texas Southwestern Medical Center, Dallas, Texas, USA

**Keywords:** LCTL, gamma Klotho, FGF, breast cancer, cancer therapy, ROS, oxidative stress, oncogene

**This article has been corrected:** The correct Keywords are given below:

***Keywords:*** LCTL; gamma Klotho; FGF; breast cancer; cancer therapy; ROS; oxidative stress; oncogene

Original article: Oncotarget. 2016; 7:2611-2628. https://doi.org/10.18632/oncotarget.6006

