# Supplementary material for: γKlotho is a novel marker and cell survival factor in a subset of triple negative breast cancers
Source: Oncotarget. 2015 Nov 9;7(3):2611–28. doi: 10.18632/oncotarget.6006 (PMC4823059; doi:10.18632/oncotarget.6006)
Supplement: Supplementary file 1 [file oncotarget-07-2611-s001.pdf]

SUPPLEMENTARY FIGURES AND TABLES

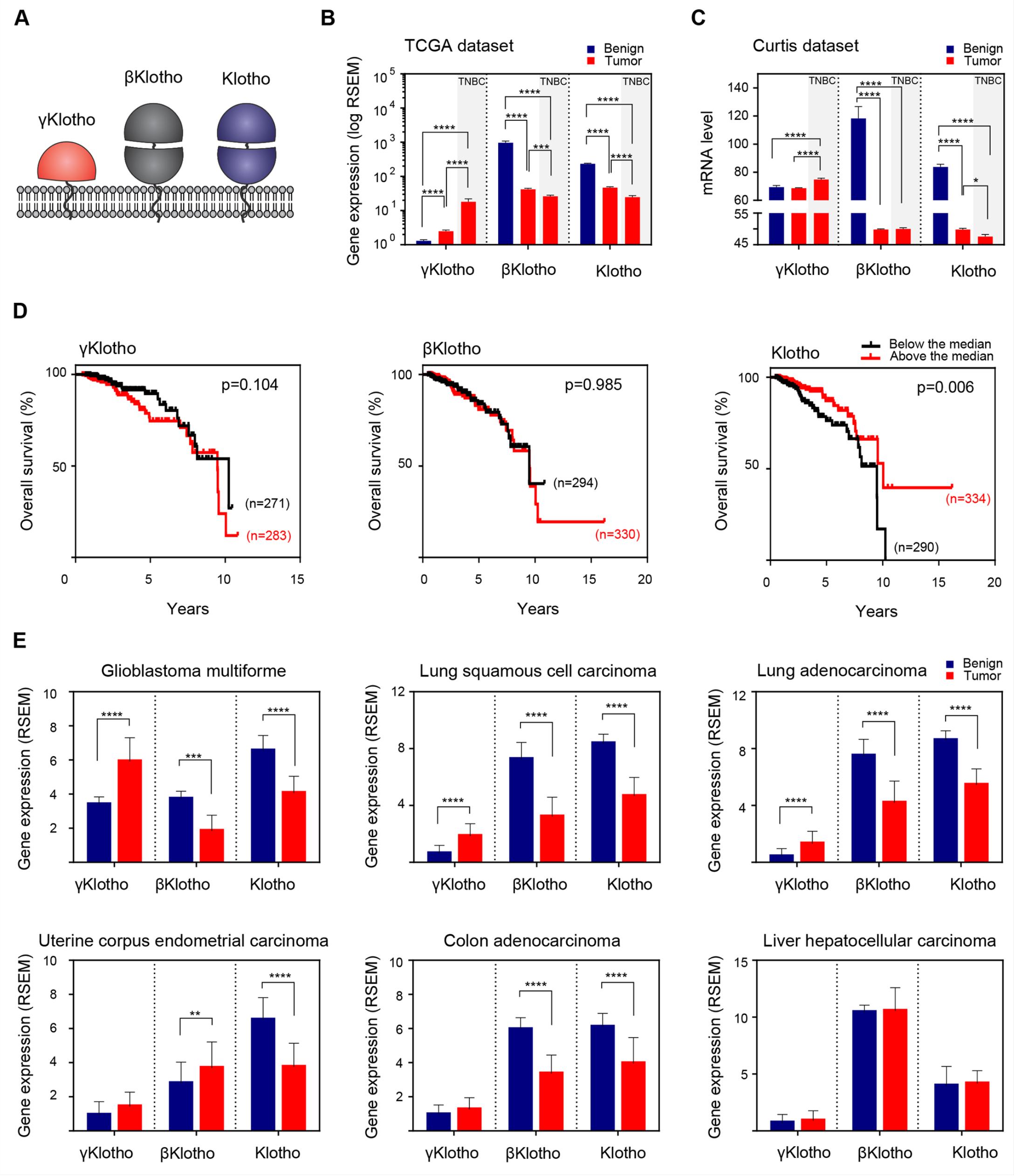

Supplementary Figure S1: Klotho proteins in cancer patients. A. Schematic representation of Klotho proteins. All three proteins are single-pass transmembrane proteins; unlike Klotho and βKlotho, γKlotho contains only a single β-glycosidase-like domain in the extracellular region. **B.** The expression of Klotho genes in breast cancers and in corresponding benign tissues. Gene expression data (RNA-Seq) from TCGA were analyzed for difference in gene expression between triple negative (n = 120) vs. non-triple negative (n = 610) breast cancer samples and benign tissue (n = 111) using one way ANOVA. **C.** The expression of Klotho genes was similarly analyzed as above in about 2,000 samples from the Curtis microarray dataset (n = 144 benign, 320 TN, 1673 nonTN), by Bonferroni post-hoc test. **D.** Association of the expression of Klotho genes with breast cancer patients overall survival based on the median for each gene. Log-rank (Mantel-Cox) test was used to calculate significance. Clinical and gene expression data were obtained by from the TCGA portal. **E.** Differential expression (RNA-Seq) of Klotho genes across TCGA primary tumors: glioblastoma multiforme (154 primary tumor, 5 normal samples), lung squamous cell carcinoma (51 matched tumor/normal samples), lung adenocarcinoma (53 matched tumor/normal samples), uterine corpus endometrial carcinoma (144 primary tumor, 24 normal samples), colon adenocarcinoma (286 primary tumor, 41 normal samples) and liver hepatocellular carcinoma (50 matched tumor/ normal samples). **$p \leq 0.01$, ***$p \leq 0.001$ ****$p \leq 0.0001$; 2-way ANOVA.

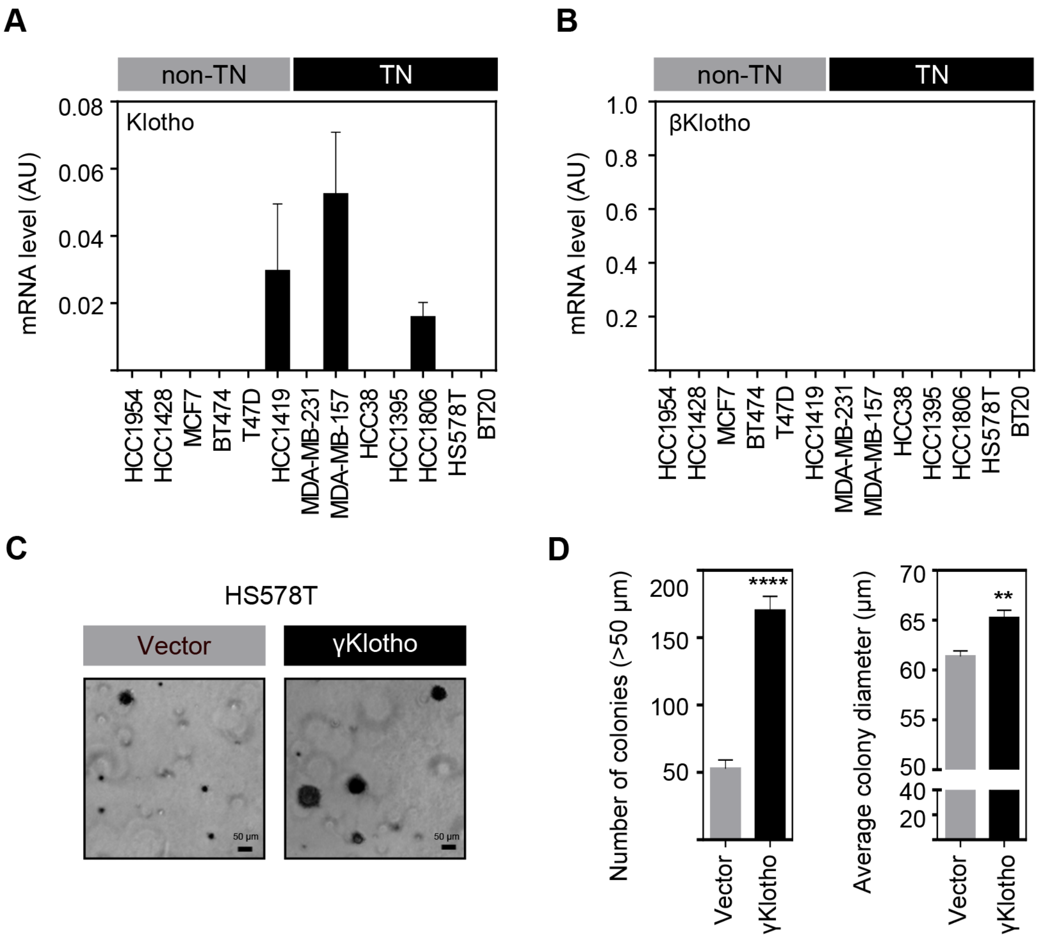

Supplementary Figure S2: γKlotho stimulates anchorage independent growth of breast cancer HS578T cells. A, B. Klotho and βKlotho mRNA expression in breast cancer cell lines was determined by qRT-PCR and normalized against Rplp0. Each bar represents the mean ± SD of two biological replicates. **C.** Images of representative soft-agar colonies in control vector or γKlotho expressing HS578T cells. **D.** Bar graph showing number (left) and average diameter (right) of soft-agar colonies in vector or γKlotho expressing HS578T cells that are larger than 50 μm in diameter. Bars demonstrate average ± SD across three independent experiments. $^{**}p \leq 0.01$, $^{****}p \leq 0.0001$; unpaired *t* test.

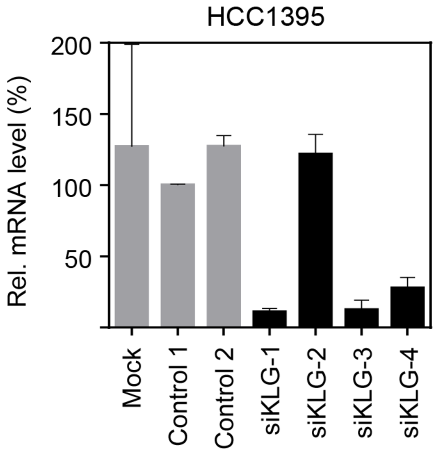

Supplementary Figure S3: The efficiency of γKlotho knockdown by siRNA transfection. HCC1395 cells were transfected with two control siRNAs or four siRNAs targeting γKlotho. Mock transfection was included as additional control. γKlotho mRNA levels were determined by qRT-PCR two days after transfection. Gene expression was normalized against Rplp0, presented values are relative to the expression in Control 1. Each bar is the mean of two biological replicates ± SD.

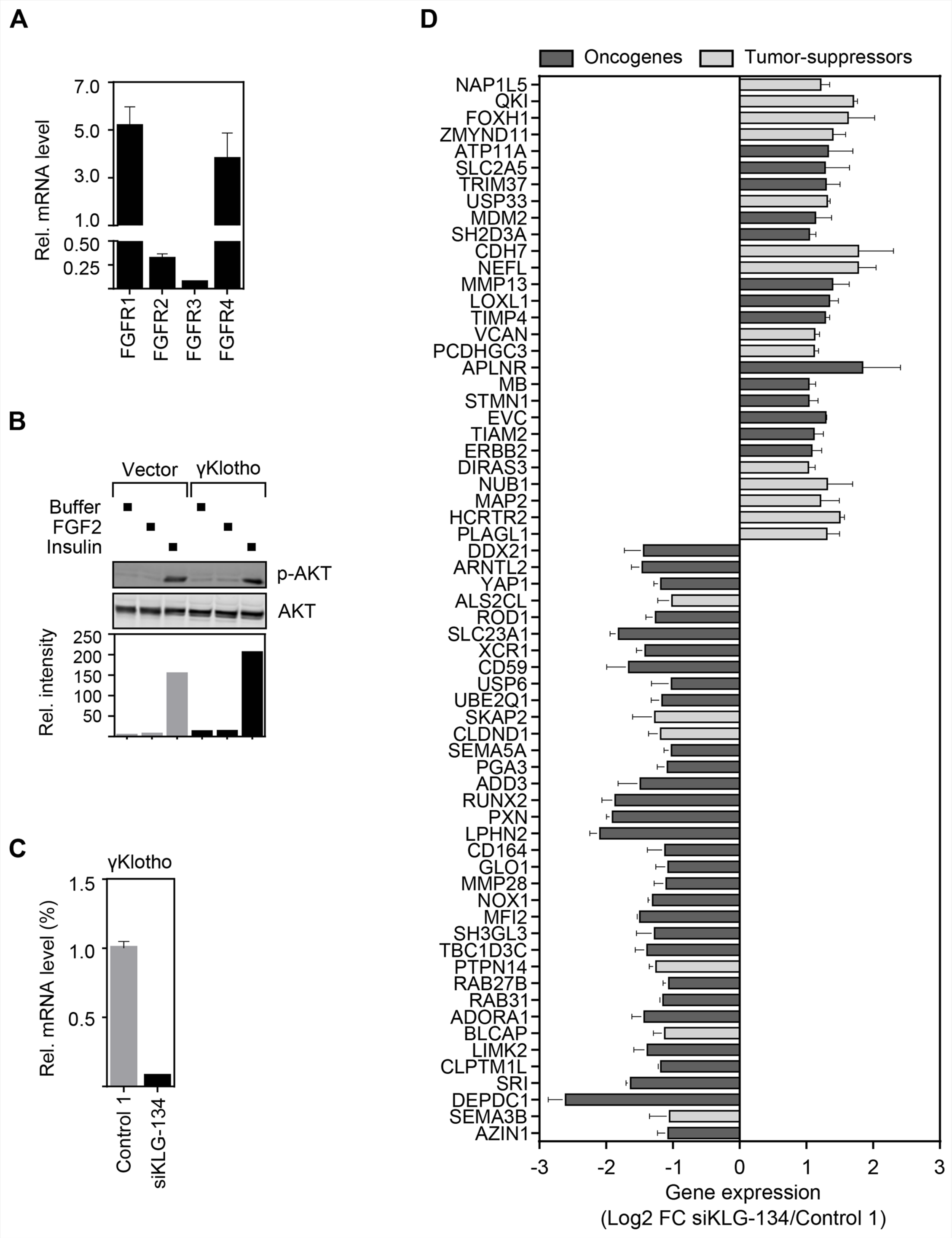

Supplementary Figure S4: Oncogenes and tumor suppressors differentially expressed after γKlotho knockdown. A. mRNA expression was determined by qRT-PCR and normalized against Rplp0. Each bar represents the mean ± SD of two biological replicates. **B.** AKT phosphorylation upon FGF2 or insulin treatment in control vector or γKlotho stably expressing MDA-MB-231 cells, measured by western blot. **C.** HCC1395 cells were transfected with control siRNA (Control 1) or a pool of siRNAs targeting γKlotho and 48 h later RNA was extracted for microarray expression analysis. Knock-down of γKlotho was confirmed by qRT-PCR. mRNA expression was normalized against Rplp0 and is presented relative to Control 1. Each bar represents the mean ± SD of two biological replicates. **D.** Levels of 64 differentially expressed genes after γKlotho ablation which were previously shown to be involved in cancer pathogenesis as oncogenes or tumor suppressor genes, as indicated. Bars represent the mean ± SD of two biological replicates.

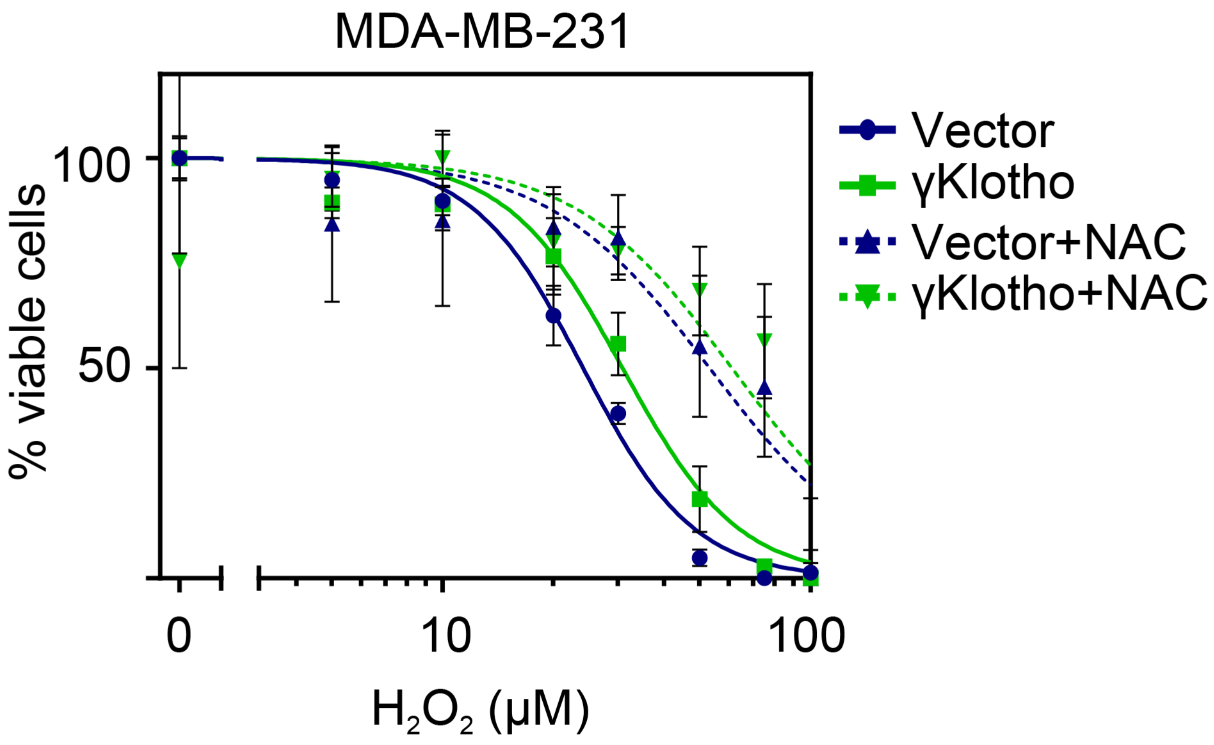

Supplementary Figure S5: γKlotho protects cancer cells against ROS toxicity. MDA-MB-231 cells stably expressing vector control or γKlotho-Flag were seeded in 96-well plates at 8000 cells/ well. 12 hours later, culture media was replaced with increasing concentrations of H_2O_2 in serum free RPMI media with or without 1 h 1 mM NAC pretreatment. 72 hours later MTS viability assay was performed. Data are normalized to the untreated controls (100% viability). Dose response curves are plotted using a non-linear regression model and IC_{50}s were determined from the fitted curves using Hill equation.

Supplementary Table S1: Patient and tumor characteristics ER, Estrogen receptor; PR, progesterone receptor; HER2, human epidermal growth factor receptor 2

Parameter	*	Number	%
**Age**	*n* = 68		
< 55		32	47.1
≥ 55		36	52.9
**Race**	*n* = 62		
Caucasian		50	80.6
Black		12	19.4
**Tumor type**	*n* = 64		
Ductal		60	93.5
Lobular		4	6.5
**Grade**	*n* = 65		
I		7	10.8
II		26	40.0
III		32	49.2
**Stage**	*n* = 68		
I		13	19.1
II		37	54.4
III		15	22.1
IV		3	4.4
**ER**	*n* = 68		
Positive		43	63.2
Negative		25	36.8
**PR**	*n* = 68		
Positive		36	52.9
Negative		32	47.1
**HER2**	*n* = 67		
Positive		11	16.4
Negative		56	83.6
**Ki6**	*n* = 67		
≤ 14%		19	28.4
> 14%		48	71.6
**p53**	*n* = 67		
≤ 10%		45	67.2
> 10%		22	32.8

*total number with known parameters

Supplementary Table S2: High levels of γKlotho correlate with high grade/stage tumors

Dataset	Grade/Stage	N	Mean expression	Stdev	*p*-value
UTSW	GI/II	33	0.00972	0.015	0.046*
	GIII	32	0.04066	0.083	
Curtis	GI	170	68.2	10.8	0.005**
	GII	775	68.6	11.8	
	GIII	957	70.9	13.7	
	S0	492	68.1	11.4	0.003**
	SI	372	71.7	12.9	
	SII	579	69.9	12.6	
	SIII	90	72.6	14.0	
	SIV	10	75.0	8.7	

*two tailed *t*-test, unpaired, unequal variance
**Spearman *p*-value, $r = 0.08$ for grade; $r = 0.076$ for stage

Supplementary Table S3: γKlotho is preferentially upregulated in TN samples*

Curtis		*n*	Percent	TCGA		*n*	Percent
	**nonTN**	**1672**	**84%**		**nonTN**	**548**	**74%**
	Downregulated	232	14%		Downregulated	77	14%
	Intermediate	1242	74%		Intermediate	450	82%
	Upregulated	198	12%		Upregulated	21	4%
	**TN**	**320**	**16%**		**TN**	**116**	**16%**
	Downregulated	35	11%		Downregulated	6	5%
	Intermediate	199	62%		Intermediate	62	53%
	Upregulated	86	27%		Upregulated	48	41%

*tumor samples were grouped into three expression levels based on +/-1 SD of the average of the cohort
